# Towards Better Understanding of the Interactions and Efficient Application of Plant Beneficial Prebiotics, Probiotics, Postbiotics and Synbiotics

**DOI:** 10.3389/fpls.2020.01068

**Published:** 2020-07-16

**Authors:** Maria Vassileva, Elena Flor-Peregrin, Eligio Malusá, Nikolay Vassilev

**Affiliations:** ^1^ Department of Chemical Engineering, Institute of Biotechnology, University of Granada, Granada, Spain; ^2^ Research Institute of Horticulture, Skierniewice, Poland

**Keywords:** plant beneficial microorganisms, prebiotics, microbial metabolites, formulation, microbiome management

## Introduction

It is well known that a gram of soil contains thousands of individual microbial taxa including bacteria, fungi, protists, oomycetes and viruses. Many of them play the main role in ecosystem functioning determining soil fertility and provide plant growth promotion and disease suppression, (van der Heijden et al., 2008; Glick, 2012; Serna-Chavez et al., 2013; Maron et al., 2018). However, after many years of chemical fertilization, soils lost their natural fertility, plant diversity and microbial richness (Huang et al., 2019). In addition, an increasing number of stress factors are observed such as salinity, alkalinity/acidity, contamination, nutrient deficiency or overload of chemical fertilizers, drought, soil erosion due to climate change, and various biotic factors (Fitzpatrick et al., 2019). The use of plant beneficial microorganisms (PBM) to mitigate these 0problems in cultivated crop production is now a common practice particularly in the modern, sustainable agriculture and in the context of increasing world population and environmental and climate concerns (Shilev et al., 2019). During the last 20–30 years, a large number of microorganisms have been isolated, characterized and tested as biofertilizers and biocontrol agents in controlled and natural conditions. The results confirmed the beneficial effect of the selected microorganisms on plant growth and health, enhancing nutrient content and improving soil properties. Now, the emphasis of the scientific activity in the field of microbial inoculants is on developing environmentally friendly and efficient microbial formulations and analyse how the introduced microorganisms affect microbial community, diversity, and the specific plant–microorganisms interactions, which determine the plant holobiome functioning (Berg et al., 2017). Therefore, at this moment, at least two major lines of research can be distinguished: the first one deals with holobiome/hologenome studies including molecular mechanisms and genetic regulation (and epigenetic mechanisms) of beneficial microbiota (Corbin et al., 2020) and, another important line of research on the process of establishing a plant beneficial microbiome includes development of efficient single or multiple microbial inoculants. A combination of pro- and postbiotics could be applied to manage and stimulate the existing beneficial microbiome.

## What is Important to Know Before Selecting a PBM?

There are many interrelated points in our understanding of the role of PBM that should be taken into consideration when designing inocula of PBM and applying them in the field. Firstly, the coexistence of all multicellular eukaryotes and microorganisms forming a holobiome and hologenome was evolutionary proved. The vast majority of recent studies including in the field of plant–microbe interactions, have confirmed the role of beneficial microorganisms in host development, metabolism, stress adaptation, and health. It appears that hosts can attract microorganisms with specific plant-beneficial characteristics (Rodrigo et al., 2017). Secondly, due to chemicalization of soils, climate and environmental changes, there is a clear decline in the soil microbial diversity and in the number of PBM: plants are less able to attract, select, and outsource their colonizers as the link between them is broken (Hardoim et al., 2015). Therefore, based on previous physical, chemical, and biological/biochemical analysis of the soil–plant system and microenvironment, we should introduce microbial inoculants composed by a single or multiple microorganism(s) (Qiu et al., 2019). Thirdly, in some cases, microbial formulated products demonstrated excellent plant growth promoting or plant protection effects under greenhouse-controlled conditions, but showed unsatisfactory results in field conditions. Moreover, some studies demonstrated reduced plant growth and increased microbial phytopathogenicity as a result of soil–plant systems inoculation with potentially beneficial microorganisms in conditions of nutrient saturation, changes in the microbial community, or environmental and plant genotype effects (Rayan and Graham, 2002; van der Heijden et al., 2008; Serna-Chavez et al., 2013; Fitzpatrick et al., 2018).

## Prebiotics, Probiotics, and Postbiotics

**Figure 1:**
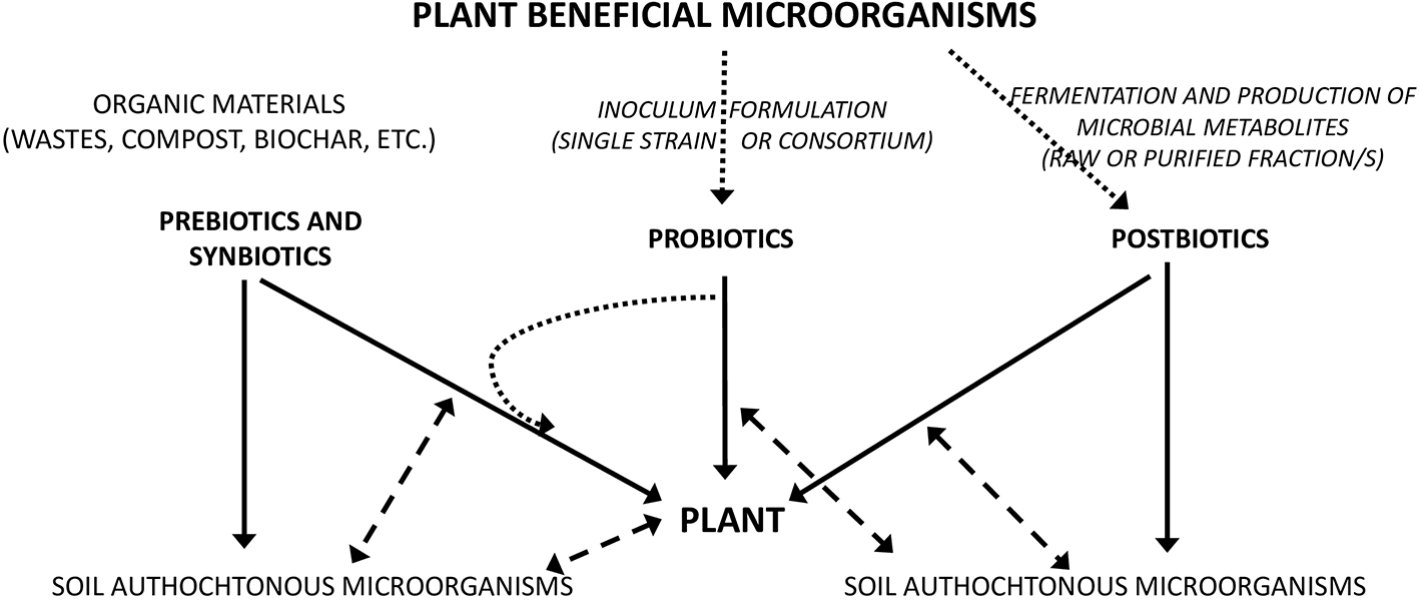
Diagram showing the three strategies for microbial management of soil–plant based on prebiotics, probiotics, and postbiotics approaches. Full lines show the direct effect, dashed lines show the interactions, dotted lines—the formulation/production processes.

Based on the above considerations, three strategies for microbial management of soil–plant systems could be selected based on prebiotics, probiotics, and postbiotics (**Figure 1**).

### Prebiotics and Synbiotics

Prebiotics are products, which improve microbial diversity and soil microbial health by promoting the growth of soil microorganisms already present within the soil–plant system. Prebiotics are natural products, normally agro-industrial wastes, including biochar, sewage sludge, compost, humus, animal manure, and chitin-bearing wastes, among others, which ameliorate (particularly in degraded soils) the soil structure, biochemical activity, and increase microbial population and diversity (Baker et al., 2011; Vassilev et al., 2013; Strachel et al., 2017). Compost and animal manure, however, can be considered as synbiotic products (Adam et al., 2016) as they contain microorganisms (some of them with beneficial properties); PBM could be additionally inoculated into the compost. Solid-state fermentation (SSF) based inoculants can also be defined as synbionts. The final SSF products are multifunctional mixtures of mineralized organic matter (with both prebiotic and carrier functions) and plant beneficial microorganism(s) (with probiotic plant growth promoting or biocontrol functions) (Vassilev and Mendes, 2018). When the probiotic microorganism is a P-solubilizing agent, the synbiotic mixture could additionally be enriched with plant available P (Shilev et al., 2019). Similar synbiotic characteristics can be observed in microbial inoculants encapsulated in natural gels in the presence of additives with beneficial microbial stimulating action (Vassilev et al., 2020).

### Probiotics

In the field of soil–plant science, probiotics are accepted as beneficial microorganisms, which exert health promoting and nutrient-mobilizing properties, as defined by Haas and Keel (2003). Particularly attractive are bacteria with high enzyme (ACC-deaminase) activity, production of phytohormones (auxins, cytokinins, gibberellins), osmolytic metabolites (e.g. trehalose, glycin betaine) (Schilev et al., 2019). These microorganisms can be found at best on the surface or within the plants (Mendes et al., 2013; Hardoim et al., 2015). Once introduced into soil, probiotics should develop a critical biomass level to exert their plant beneficial traits. As this process is highly dependent on the soil–plant characteristics and environmental conditions, it seems difficult for a given single microorganism or a microbial consortium to reach this critical cell number (Woo and Pepe, 2018). Therefore, after a long period of studies on isolation, selection, and characterization of PBM, research scientists are focused on development of economic biotechnological processes for biomass/spores production and formulation that will solve the above problems (Bashan et al., 2016; Parnell et al., 2016; Vassilev and Mendes, 2018). Formulated products can be liquid or solid and should fulfil a number of requirements, the most important of which are to demonstrate high colonizing effectiveness and competitiveness, and increase plant nutrition and health status (Malusa and Vassilev, 2014). One of the most promising formulation techniques is the encapsulation in macro- and micro-beads of polysaccharides which guarantees a continuous deliver of the inoculant into soil preventing the effect of soil and environmental stress factors including indigenous microbial community (Bashan et al., 2016; Qiu et al., 2019). However, a simple gel-entrapment is not sufficient to ensure economical advantages and desired agronomic impact of the formulates (Vassilev et al., 2020). Double/multiple inoculants combined with biostimulants and other additives including seeds (all-in-one smart bio-formulates) should be developed to complete with the traditional chemical fertilizers (Vassilev et al., 2015; Trivedi et al., 2017). Another option, to avoid problems during each phase within production, formulation, storage, and establishment/action of the PBM in soil, is to use their plant beneficial metabolites (postbiotics).

### Postbiotics

Postbiotics are metabolic derivatives of PBM, which exert specific, growth promoting and/or biocontrol, effects on plants thus avoiding the risks associated with applying microbial cells. Specific examples of such metabolite include phytohormones, volatiles, and quorum-sensing compounds (Schikora et al., 2016). Which are the risks of using microorganisms in soil–plant systems? Wrong formulation procedures without osmoprotectants, UV-protectors, fillers with nutrient value, and other plant benefiting additives usually provoke inconsistent results under field conditions (Bashan et al., 2016; Vassilev et al., 2020). Further risks include various abiotic and biotic factors, which affect the rate of microbial colonization, the presence of other, more competent, components of the microbial population, the level of plant needs and capacity to attract and feed beneficial microorganism (Fierer, 2017). It is important to note that the protocols for field applications of PBM are not assuring that they will find their niche of establishing and function. Moreover, it is yet not clearly known what kind of metabolites the introduced microorganisms will release in the soil–plant system. This complex set of conditions determines the rate of survival of the inoculants and the performance of their target functions (Kaminsky et al., 2019). Analysing all these aspects, it appears that endophytic microorganisms are better protected from adverse environmental conditions and, in addition, more efficient functionally (Santoyo et al., 2017).

Shall we apply cell-free liquids containing specific or complex metabolites produced by the PBM during fermentation under controlled conditions? There are two options in developing such kind of biotechnological products. Using cell-free fermentation broth liquids without further downstream operations for separation/purification of specific metabolites is the most economic option and, in some cases mixtures of different microbial cultures demonstrate higher potential even after autoclaving (Mendes et al., 2017; Hussain et al., 2020). Well-established and easy to perform immobilized cell technology methods can be applied to repeatedly/continuously use the metabolic activity of the microorganisms (Kautola et al., 1990), producing plant growth promoting or biocontrol compounds in repeated-batch or continuous fermentation mode thus making the whole process more attractive economically (Vassilev et al., 2017; Mishra and Arora, 2018).

Another approach includes operations such as fragmentation and further use of extracts of the microbial mass or isolation of specific metabolites from the fermentation liquid. However, the application of specific metabolites in soil should be assessed carefully, bearing in mind that in the rhizosphere there is a great variety of microbial and plant metabolites involved in a wide number of interrelated cooperative or antagonistic actions (Besset-Manzoni et al., 2018). Therefore, before applying plant beneficial metabolites directly after the fermentation production process or in purified form, formulation operations should be performed to ensure their efficient release into soil. Encapsulation and nano-encapsulation of microbial metabolites was reported as an effective tool in enhancing proliferation of shoots and rooting (Pour et al., 2019). In this case, the inclusion of carbon nanotubes and SiO nanoparticles in the alginate-gelatin nanocapsules increased the overall beneficial effect of the formulated cell-free product. Nano-formulations by encapsulation are expected to enhance the metabolic stability of the microbial metabolites but their cost-effectiveness can be increased if the principles of the precision agriculture are applied (Duhan et al., 2017).

## Concluding Remarks

Production and application of PBM is now one of the most promising fields of research. The period of searching for easy to cultivate soil microorganisms, their characterization, and testing in controlled conditions was replaced by another one with studies on novel, more efficient and economic fermentation mode of production and formulations. Co-cultivation and formulation of compatible PBM and inclusion of various additives in the formulations become fundamental part of the overall production technology (Vassilev et al., 2014; Vassilev et al., 2015; Vassilev et al., 2020). Another, pivotal point of the new approach to understand and manage the functional and genetic role of soil microorganisms in the soil–plant systems, is the comparison between human gut microbiome and plant microbiome (Adam et al., 2016). Following the human gut example, new strategies for exploitation of PBM appeared based on prebiotic, probiotic, synbiotic, and postbiotic products. A previous analysis of soil physical/chemical characteristics, microbial community dynamics along the plant growth and depending on the climatic specificity is a part of the overall assessment on which approach will be most efficient. Here, we consciously do not discuss, but should mention, other important issues such as how to control the plant capability of attracting useful microorganisms, the role of core and hub microbiota (Toju et al., 2018), and development of multi-omics tools and interdisciplinary (or artificial intelligence) approaches of management of all soil–microbe spatio-temporal complex data (Aleklett et al., 2017). The advancement in the field of PBM is substantial but there are still largely unexplored options for “biotics” therapeutic treatment of soils and biotechnological optimization of microbiome functioning in agro-soil systems bearing in mind their extreme complexity (Fierer, 2017).

## Author Contributions

MV and NV designed and drafted the work. EM and EF-P contributed to the revision of the manuscript.

## Funding

This work was supported by the project EXCALIBUR funded from the European Union’s Horizon 2020 research and innovation programme under grant agreement No. 817946.

## Conflict of Interest

The authors declare that the research was conducted in the absence of any commercial or financial relationships that could be construed as a potential conflict of interest.

## References

[B1] Adam E. Groenenboom A. E. Kurm V. Rajewska M. Schmidt R. Tyc O. (2016). Controlling the Microbiome: Microhabitat Adjustments for Successful Biocontrol Strategies in Soil and Human Gut. Front. Microbiol. 7, 1079. 10.3389/fmicb.2016.01079 27468279 PMC4942455

[B2] Aleklett K. Kiers E. T. Ohlsson P. Shimizu T. S. Caldas V. E. Hammer E. C. (2017). Build your own soil: exploring microfluidics to create microbial habitat structures. ISME J. 12 (2), 312–319. 10.1038/ismej.2017.184 29135971 PMC5776464

[B3] Baker L. R. White P. M. Pierzynski G. M. (2011). Changes in microbial properties after manure, lime, and bentonite application to a heavy metal-contaminated mine waste. Appl. Soil Ecol. 48 (1), 1–10. 10.1016/j.apsoil.2011.02.007

[B4] Bashan Y. de-Bashan L. E. Prabhu S. R. (2016). “Superior polymeric formulations and emerging innovative products of bacterial inoculants for sustainable agricultura and the environment,” in Agriculturally Important Microorganisms. Eds. Singh H. B. Sarma B. K. Keswani C. (Singapur: Springer), 15–46.

[B5] Berg G. Köberl M. Rybakova D. Müller H. Grosch R. Smalla K. (2017). Plant microbial diversity is suggested as the key to future biocontrol and health trends. FEMS Microbiol. Ecol. 93 (5), fix050. 10.1093/femsec/fix050 28430944

[B6] Besset-Manzoni Y. Rieusset L. Joly P. Comte G. Prigent-Combaret C. (2018). Exploiting rhizosphere microbial cooperation for developing sustainable agriculture strategies. Environ. Sci. Poll. Res. 25, 29953–29970. 10.1007/s11356-017-1152-2 29313197

[B7] Corbin K. R. Bolt B. Rodriguez-Lopez C. M. (2020). Breeding for beneficial microbial communities using epigenomics. Front. Microbiol. 11:, 937. 10.3389/fmicb.2020.00937 32477316 PMC7242621

[B8] Duhan J. S. Kumar R. Kumar N. Kaur P. Nehra K. Duhan S. (2017). Nanotechnology: The new perspective in precision agriculture. Biotechnol. Rep. 15, 11–23. 10.1016/j.btre.2017.03.002 PMC5454086 28603692

[B9] Fierer N. (2017). Embracing the unknown: disentangling the complexities of the soil microbiome. Nat. Rev. Microbiol. 15, 579–590. 10.1038/nrmicro.2017.87 28824177

[B10] Fitzpatrick C. R. Copeland J. Wang P. W. Guttman D. S. Kotanen P. M. Johnson M. T. J. (2018). Assembly and ecological function of the root microbiome across angiosperm plant species. Proc. Natl. Acad. Sci. U. S. A. 115, 1157–1165. 10.1073/pnas.1717617115 PMC5819437 29358405

[B11] Fitzpatrick C. R. Mustafa Z. Viliunas J. (2019). Soil microbes alter plant fitness under competition and drought. J. Evol. Biol. 00, 1–13. 10.1111/jeb.13426 30739360

[B12] Glick B. (2012). Plant growth-promoting bacteria: Mechanisms and applications. Scientifica 2012, 963401. 10.6064/2012/963401 24278762 PMC3820493

[B13] Haas D. Keel C. (2003). Regulation of antibiotic production in root-colonizing *Pseudomonas* spp. and relevance for biological control of plant disease. Ann. Rev. Phytopathol. 41, 117–153. 10.1146/annurev.phyto.41.052002.095656 12730389

[B14] Hardoim P. R. van Overbeek L. S. Berg G. Pirttilä A. M. Compant S. Campisano A. (2015). The hidden world within plants: ecological and evolucionary considerations for defining functioning of microbial endophytes. Microbiol. Mol. Biol. Rev. 79, 293–320. 10.1128/MMBR.00050-14 26136581 PMC4488371

[B15] Huang R. Mcgrath S. Hirsch P. Clark I. Storkey J. Wu L. (2019). Plant–microbe networks in soil are weakened by century-long use of inorganic fertilizers. Microb. Biotechnol. 12, 1464–1475. 10.1111/1751-7915.13487 31536680 PMC6801139

[B16] Hussain T. Haris M. Shakeel A. Ahmad G. Khan A. A. Khan M. A. (2020). Bio-nematicidal activities by culture filtrate of *Bacillus subtilis HussainT*-*AMU*: new promising biosurfactant bioagent for the management of Root Galling caused by *Meloidogyne incognita* . Vegetos. 33, 298–238. 10.1007/s42535-020-00099-5

[B17] Kaminsky L. M. Trexler R. V. Malik R. J. Hockett K. L. Bell T. H. (2019). The inherent conflicts in developing soil microbial inoculants. Trends Biotechnol. 37, 140–151. 10.1016/J.TIBTECH.2018.11.011 30587413

[B18] Kautola H. Vassilev N. Linko Y. Y. (1990). Continuous itaconic acid production by immobilized biocatalysts. J. Biotechnol. 13, 315–323. 10.1016/0168-1656(90)90079-Q 1366363

[B19] Malusá E. Vassilev N. (2014). A contribution to set a legal framework for biofertilisers. Appl. Microbiol. Biotechnol. 98, 6599–6607. 10.1007/s00253-014-5828-y 24903811 PMC4108841

[B20] Maron P.-A. Sarr A. Kaisermann A. Lévêque J. Mathieu O. Guigue J. (2018). High microbial diversity promotes soil ecosystem functioning. Appl. Environ. Microbiol. 84, e02738–e02717. 10.1128/AEM.02738-17 29453268 PMC5930326

[B21] Mendes R. Garbeva P. Raaijmakers J. M. (2013). The rhizosphere microbiome: significance of plant beneficial, plant pathogenic, and human pathogenic microorganisms. FEMS Microbiol. Rev. 37, 634–663. 10.1111/1574-6976.12028 23790204

[B22] Mendes G. Galvez A. Vassileva M. Vassilev N. (2017). Fermentation liquid containing microbially solubilized P significantly improved plant growth and P uptake in both soil and soilless experiments. Appl. Soil Ecol. 117, 208–211. 10.1016/j.apsoil.2017.05.008

[B23] Mishra J. Arora N. K. (2018). Secondary metabolites of fluorescent pseudomonads in biocontrol of phytopathogens for sustainable agriculture. Appl. Soil Ecol. 125, 35–45. 10.1016/j.apsoil.2017.12.004

[B24] Parnell J. J. Berka R. Young H. A. Sturino J. M. Kang Y. Barnhart D. M. (2016). From the lab to the farm: an industrial perspective of plant beneficial microorganisms. Front. Plant Sci. 7, 1110. 10.3389/fpls.2016.01110 27540383 PMC4973397

[B25] Pour M. M. Saberi-Riseh R. Mohammadinejad R. Hosseini A. (2019). Nano-encapsulation of plant growth-promoting rhizobacteria and their metabolites using alginate-silica nanoparticles and carbón nanotube improves UCB1 Pistachio Micropropagation. J. Microbiol. Biotechnol. 29, 1096–1103. 10.4014/jmb.1903.03022 31091866

[B26] Qiu Z. Egidi E. Liu H. Kaur S. Singh B. K. (2019). New frontiers in agricultura productivity: Optimized microbial inoculants and in situ microbiome engineering. Biotechnol. Adv. 37, 107371. 10.1016/j.biotechadv.2019.03.010 30890361

[B27] Rayan M. H. Graham J. H. (2002). Is there a role for arbuscular mycorrhizal fungi in production agricultura? Plant Soil 244, 263–271. 10.1007/978-94-017-1284-2_26

[B28] Rodrigo A. Rogers M. Bohlig B. (2017). The evolutionary value of helpful microbes: A response to Shapira. Trends Ecol. Evol. 32, 84–85. 10.1016/j.tree.2016.11.002 27915201

[B29] Santoyo G. Hernández-Pacheco C. Hernández-Salmerón J. Hernández-León R. (2017). The role of abiotic factors modulating the plant-microbe-soil interactions: toward sustainable agriculture. A review. Span. J. Agric. Res. 15 (1), e03R01, 15 pages. 10.5424/sjar/2017151-9990

[B30] Schikora A. Schenk S. T. Hartmann A. (2016). Beneficial effects of bacteria-plant communication based on quorum sensing molecules of the N -acyl homoserine lactone group. Plant Mol. Biol. 90, 605–612. 10.1007/s11103-016-0457-8 26898296

[B31] Serna-Chavez H. M. Fierer N. van Bodegom P. M. (2013). Global drivers and patterns of microbial abundance in soil. Global Ecol. Biogeog. 22, 1162–1172. 10.1111/geb.12070

[B32] Shilev S. Azaizeh H. Vassilev N. Georgiev D. Babrikova I. (2019). “Interactions in soil-microbe-plant system: adaptation to stressed agriculture,” in Microbial Interventions in Agriculture and Environment. Eds. Singh D. Gupta V. Prabha R. (Singapore: Springer), 131–171. 10.1007/978-981-13-8391-5_6

[B33] Strachel R. Wyszkowska J. Baćmaga M. (2017). The Role of Compost in Stabilizing the Microbiological and Biochemical Properties of Zinc-Stressed Soil. Water Air Soil Pollut. 228, 349. 10.1007/s11270-017-3539-6 28890580 PMC5569127

[B34] Toju H. Peay K. G. Yamamichi M. Narisawa K. Hiruma K. Naito K. (2018). Core microbiomes for sustainable agroecosystems. Nat. Plants 4, 247–257. 10.1038/s41477-018-0139-4 29725101

[B35] Trivedi P. Schenk P. M. Wallenstein M. D. Singh B. K. (2017). Tiny microbes, big yields: enhancing food crop production with biological solutions. Microb. Biotechnol. 10, 999–1003. 10.1111/1751-7915.12804 28840959 PMC5609239

[B36] van der Heijden M. G. A. Bardgett R. D. van Straalen N. M. (2008). The unseen majority: soil microbes as drivers of plant diversity and productivity in terrestrial ecosystems. Ecol. Lett. 11, 296–310. 10.1111/j.1461-0248.2007.01139.x 18047587

[B37] Vassilev N. Mendes G. (2018). “Solid-state fermentation and plant beneficial microorganisms,” in Current Developments in Biotechnology and Bioengineering, Current Advances in Solid-State Fermentation. Eds. Pandey A. Larroche C. H. Soccol C. (Amsterdam: Elsevier), 402–416.

[B38] Vassilev N. Martos E. Mendes G. Martos V. Vassileva M. (2013). Biochar of animal origin: a sustainable solution of the high-grade rock phosphate scarcity. J. Sci. Food Agric. 93, 1799–1804. 10.1002/jsfa.6130 23504602

[B39] Vassilev N. Mendes G. Costa M. Vassileva M. (2014). Biotechnological tools for enhancing microbial solubilization of insoluble inorganic phosphates. Geomicrobiol. J. 31, 751–763. 10.1080/01490451.2013.822615

[B40] Vassilev N. Vassileva M. Lopez D. Martos V. Reyes A. Maksimivich I. (2015). Unexploited potential of some biotechnological techniques for biofertilizer production and formulation. Appl. Microbiol. Biotechnol. 99, 4983–4996. 10.1007/s00253-015-6656-6654 25957155

[B41] Vassilev N. Eichler-Löbermann B. Flor-Peregrin E. Martos V. Reyes A. Vassileva M. (2017). Production of a potential liquid plant bio-stimulant by immobilized *Piriformospora indica in* repeated-batch fermentation process. AMB Express 7, 106. 10.1186/s13568-017-0408-z 28549373 PMC5445041

[B42] Vassilev N. Vassileva M. Martos V. Garcia del Moral L. F. Kowalska J. Tylkowski B. (2020). Formulation of Microbial Inoculants by Encapsulation in Natural Polysaccharides: Focus on Beneficial Properties of Carrier Additives and Derivatives. Front. Plant Sci. 11, 270. 10.3389/fpls.2020.00270 32211014 PMC7077505

[B43] Woo S. L. Pepe O. (2018). Microbial Consortia: Promising Probiotics as Plant Biostimulants for Sustainable Agriculture. Front. Plant Sci. 9, 1801. 10.3389/fpls.2018.01801 30564264 PMC6288764

